# Eucalyptus Globulus

**Published:** 1881-06

**Authors:** 


Eucalyptus Globulus. Its Value in Chronic Diseases of the Stomach and Bowels.—Inasmuch as the experience of the past four years in the use of the above drug has resulted very successfully in these classes of chronic disease, it occurred to me to give my professional brethren the benefit of my experience, and encourage them to give it a trial in such cases, for it is only by an unprejudiced and cautious accumulation of experience that the real value of a therapeutic agent can be estimated. Of course I do not regard it as a specific in any case where it may seem to be indicated, for all the details as to diet and general regimen must be attended to in these diseases.

Out of some thirty cases the following fully illustrates its therapeutical value. Mr. B., æt. 66, presented himself with a history of stomach difficulty for fifteen years, and several times seemed to be near his end, with all the symptoms of malignant ulceration. His history was as follows: Vomiting of quantities of blood from time to time; the stomach seemed to be distended by a sour barmy fluid, which was relieved by vomiting; during such times life seemed to be prolonged by careful attention to diet.

Various physicians had prescribed the usual routine of treatment, but without avail, at last he regarded himself as beyond the reach of all remedies. I at once put him on the tinct. of eucalyptus in doses of ʒj, twice a day, and ordered him to report in a few weeks. After the first day he noticed an improvement, which continued steadily, until cured at the end of three months, and during all that time he had scarcely even a threatening of those painful attacks which formerly occurred twice a week.

In a class of cases of symptoms of ulcers of the stomach, threatening perforation, I have found that strict regimen and light diet, conjoined with the use of the drug, exempted the patient from the recurrence of attacks, which past experience had led them to always anticipate.—Chas. Jas. Fox, A.M., M.D., in the *Medical Bulletin.*

